# Albuminuria and Life Assurance

**Published:** 1890-08-30

**Authors:** 


### The Practitioner's Mirror and Retrospect.

[*The Editor will be glad to receive offers of co-operation and contributions from members of the profession. There is no doubt that much valuable material full of interest and instruction is at present lost to science. This is largely so in the case of* (1) *the younger and less-known members of the hospital staff* (2) *those connected with cottage hospitals, and* (3) *the great body of medical practitioners not attached to any institution. The co-operation of all is cordially invited to enable the Editor to make* The Hospital *of the utmost possible use and value to the profession. Specialists willing to review books on their special subjects are invited to communicate this fact at once. All letters should be addressed to* The Editor, The Lodge, Porchester Square, London, W.]

### MEDICINE.

### ALBUMINURIA AND LIFE ASSURANCE.

At the present time there is a somewhat divided view of albuminuria. Some maintain that there is no such thing as physiological albuminuria, but that it is pathological in every case, even although a mere trace only be present. And there is said to be danger, that a long continued filtration of albumen among the gland cells of the kidneys, may by degrees so disorganize them as to render them incapable of performing their functions. Cases, however, have been recorded by Dr. G. Johnson, and others, of albumen passing for years without resulting in serious disease of the kidneys. On the other hand, serious disease of the kidneys may be present without the appearance of albumen in the urine. Even granular degeneration of the kidneys has existed without the symptom of albumen being present. Dr. Costa relates a case where there was marked deposit for some time of crystals of oxalate of lime, accompanied by tube-casts of the hyaline and waxy varieties, and yet no albumen was detected. Dr. Mahomed demonstrated by *post mortem* examinations at Guy's Hospital, that disease of the kidneys may be present without albumen, the only indications of kidney disease being increased tension of the pulse, a heaving left ventricle, and increased quantity of urine. Such conditions have been termed the pre-albuminuric stage of Bright's disease. Some time ago the examiner for a large British life insurance company stated he relied on heat and nitric acid as the test for albumen. But should this test be still relied upon, if there are more delicate ones? Dr. W. H. Washburn, of Milwaukee, in an article on "Albuminuria in the Apparently Healthy" (*Philadelphia Medical News*) objects to this, and recommends picric acid test. He says that between the dates, April 1st, 1888, and February 1st, 1890, he examined 338 applicants for life insurance, in which an examination of the urine was required. All the applicants were apparently healthy, but albumen was found in 5·91 per cent. Dr. Washburn was able to follow up three of the latter cases. In the first case, the facts that the specific gravity was normal, that the microscope revealed oxalate of lime crystals, and that the patient suffered from flatulent indigestion, led to the diagnosis of albuminuria due to the presence of sharp irritating crystals, the formation of the crystals being attributed to digestive disburbances. The second case was that of a man whose urine at the first examination showed a large amount of albumen, and afterwards none. This person had noticed that whenever he had a cold his urine was passed in smaller quantities, and oftener than normal. It has, however, long been noticed that some persons pass albumen when they take cold. The third case was that of a man of sedentary habits. From June to November many examinations of the urine were made and albumen always detected, but the specific gravity was normal. Commencing, "lithæmic nephritis" was diagnosed, lithia water was prescribed, and the diet restricted. The person then took to athletics and the albumen disappeared. "The moment increased muscular activity resulted in the conversion of a larger amount of albuminous blood constituents into urea, so that there was less to be converted by the renal epithelium, albumen disappeared from the urine."

In a recent number of the *Lancet* Dr. Thomas Fraser, of Edinburgh, remarks it would be an advantage if one system of testing were invariably adopted, in which the re-agent employed is sufficiently delicate to distinguish small quantities of albumen, and is at the same time free from objections. He represents the advantage of boiling a small quantity of the urine in a test tube, then adding a drop or two of concentrated nitric acid, and again boiling. If the proportion of albumen exceeds one-eighth of the urine after it has stood at rest for 24 hours, the person should be rejected. If the urine contains a smaller quantity than one-eighth, the person should be rejected if there be enlargement of heart, increase of arterial tension, increase in the quantity of urine, unduly low specific gravity (1010 being the lowest consistent with health), a sediment containing epithelial, fatty, or granular casts, hyaline tube casts, dropsy, history of lead poisoning, scarlet fever after twelve years of age, evidence of gout, or syphilis. If the urine contains a smaller quantity of albumen, unaccompanied with any evidence of important disease of heart, the urine should be examined on several consecutive days, a sample of the whole urine of the twenty-four hours being used. If no albumen be found on any subsequent examination, the patient, Dr. Fraser thinks, may be accepted at ordinary rates. If albumen is found, the person should be delayed for six months. Now we do not think that the majority of medical men will agree altogether with Dr. Fraser. Dr. William Roberts gives the conditions under which albumen appears in the urine, as during acute and chronic Bright's disease, pregnancy, febrile and inflammatory diseases, impediments to the circulation of blood, from emphysema, heart disease, etc.; during scurvy, pyæmia, hospital gangrene, etc., during saturnine intoxication, during certain functional disturbances, and during certain nervous disturbances, as epilepsy, tetanus, etc. It may be presumed that albumen passing in connexion with any of the above conditions, excepting pregnancy and functional disturbances, would be a bar to life assurance at ordinary rates. If albumen were not discovered after pregnancy, the condition during pregnancy would not be prohibitive. So called functional albuminuria has received much attention from Moxon, Saundby, and others, but the causes are not yet fully understood. It may follow different kinds of food, or chill, or muscular exertion, although as mentioned above, Dr. Washburn shows that it may disappear under exercise. The functional character of the disorder may be recognised from its intermittent occurrence. In some cases albumen is only found after meals, in others at intervals of two or three days. Elements in the diagnosis are also the presence or absence of other accompaniments of Bright's disease, such as high arterial tension and cardiac hypertrophy. But some authorities, as Dr. Dukes and Dr. G. Johnson, are inclined to think functional albuminuria may end in Bright's disease, and in this we agree. A recent author remarks, that when albumen is found in the urine, the important point to decide is whether it indicates organic disease in the kidneys or not. This is determined by the temporary or persistent duration of the albuminuria, by the quantity of the albumen, by the presence or absence of renal deposits, by the presence or absence of any other disease which will account for albuminuria. A persistent duration of albumen, a greater proportion of albumen, increase of quantity of urine, and low density, casts and renal epithelium, no other disease, are characteristic of kidney affection. If it be admitted that the passage of albumen may not necessarily point to a present diseased condition of the kidneys, and we think this may be allowed, still the passage of albumen is a morbid process. Normal urine does not contain it. And when it appears some organ must be in fault. The late Dr. Murchison considered an over-worked liver was often a cause. Others refer it to alterations in the condition of the blood from digestive derangements. Whatever may be the cause, there is, as we think, always danger to the structure of the kidney by the passage of an abnormal urine.

Notwithstanding Dr. Washburn's advice to use picric acid, we believe that heat and nitric acid are the best tests yet discovered. Various other tests have been proposed, and some of them are perhaps more delicate, but none of them so free from sources of fallacy. But when using the boiling test it is important to attend to the due acidulation of the urine, for if the urine is alkaline albumen is not coagulated. The quantitive estimation is also of importance. For practical purposes there is no better procedure than boiling the urine with a drop or two of acetic acid. The albumen presently sinks, and may be estimated by numbers, as one-half or one-twelfth. There are other more elaborate processes, such as boiling; throwing on a weighed filter; washing; drying; and weighing. But this takes time, and requires accurate manipulation. Then there is the minimetric or dilution method proposed by Dr. W. Roberts, which also takes time, and requires special apparatus. It would certainly be desirable for life assurance societies to come to some general arrangement as to albumen in the urine, and as to the tests which should be used. Most life insurance societies make albuminuria a definite ground for rejection. We know, however, that there is albuminuria of a transient kind; also that there are cases where the albumen is only present at certain times of the twenty-four hours. But, in our opinion, although albumen may not indicate organic disease, there is quite sufficient in the fact of albumen being passed to render the case doubtful. At the meeting of the "British Medical Association," at Leeds, a discussion took place on this matter. The majority of those who took part in it were opposed to the acceptance of cases of albuminuria on any terms. Even among those who would accept some cases of albuminuria there was much difference of opinion as to the cases which should be accepted. If it be correct that the passage of albumen may eventually cause alterations of kidney structure it does not much matter whether the albumen is caused by cold, or by digestive derangements, or by mental work, or anything else. But the point has yet to be cleared whether or not the passage of albumen does cause alterations of structure.

